# Efficacy of Resin Infiltrants in Non-Cavitated Occlusal Carious Lesions: A Systematic Review

**DOI:** 10.3390/jcm15031310

**Published:** 2026-02-06

**Authors:** Samille Biasi Miranda, Rodrigo Barros Esteves Lins, Julia Almeida Maciel da Silveira, Caroline de Farias Charamba Leal, Caroline Mathias Carvalho de Souza, Helene Soares Moura, Lorena Gomes Guimarães, Priscila Prosini, Marcos Antonio Japiassú Resende Montes

**Affiliations:** 1 Department of Dental Materials, Faculty of Dentistry, University of Pernambuco, Recife 50100-130, PE, Brazil; samille.biasi@upe.br (S.B.M.); julia.almeidamaciel@upe.br (J.A.M.d.S.); caroline.charamba@upe.br (C.d.F.C.L.); lorena.gguimaraes@upe.br (L.G.G.); priscila.prosini@upe.br (P.P.); 2 School of Dentistry, Federal University of Alagoas, Maceió 57072-900, AL, Brazil; rodrigo.lins@foufal.ufal.br; 3 School of Dentistry, Faculty of Technology and Sciences, Salvador 41741-590, BA, Brazil; mathias.souza1@ftc.edu.br; 4 School of Dentistry, State University of Paraíba, Araruna 58429-500, PB, Brazil; helene.smoura@gmail.com

**Keywords:** resin infiltrants, non-cavitated occlusal carious lesions, dental caries, dental sealants, systematic review

## Abstract

**Objectives**: To evaluate the efficacy of resin infiltrants (RIs) in controlling non-cavitated occlusal carious lesions (NCOCLs) in primary and permanent teeth. **Methods**: This systematic review followed PRISMA guidelines. Randomized clinical trials (RCTs) and in vitro/ex vivo studies comparing RI with placebo or other materials were included. Searches were conducted in five databases and gray literature up to December 2025. Risk of bias was assessed using the RoB 2.0 tool for RCT and an adapted instrument for in vitro/ex vivo studies. Certainty of evidence was evaluated using the GRADE tool, and data were synthesized qualitatively. **Results**: Eight studies were included, of which only two were RCTs, and six were in vitro and ex vivo studies. RCTs showed reduced caries progression in infiltrant-treated groups compared with controls, with efficacy comparable to conventional sealants. In vitro studies demonstrated improved resin penetration and sealing ability with optimized protocols. Risk of bias ranged from low to moderate. Certainty of clinical evidence was low, primarily due to the limited number of RCTs and methodological limitations. **Conclusions**: RIs may be effective in managing NCOCLs, with performance comparable to conventional preventive approaches. However, the limited number of clinical trials and short follow-up periods reduce the strength of the evidence. Long-term clinical studies are needed to confirm the sustained effectiveness and durability of RIs.

## 1. Introduction

Dental caries is a biofilm-sugar-dependent disease characterized by an ecological imbalance within the biofilm induced by frequent exposure to fermentable sugars. This imbalance leads to multiple episodes of pH drop and demineralization, which, when exceeding the capacity for remineralization, result in cumulative mineral loss clinically manifested as a carious lesion [1,2]. It is the most prevalent untreated oral condition worldwide, affecting billions of people [3].

The earliest clinical signs of caries appear as non-cavitated lesions, also known as initial lesions or white-spot lesions [4,5]. When the caries process is not arrested by controlling etiological factors or by implementing an appropriate intervention, these lesions may progress to cavitated forms due to increased mineral loss [6]. Early detection is essential in minimally invasive dentistry, which aims to halt lesion progression and promote remineralization, thereby reducing the need for more invasive restorative procedures [6,7].

For early occlusal lesions, pit-and-fissure sealants remain the most widely used minimally invasive approach [8], although they present limitations related to penetration ability and marginal sealing of the lesion [9]. In light of these limitations, studies have evaluated the use of resin infiltrants as a potential clinical alternative for occlusal surfaces, aiming to determine whether they can overcome the shortcomings of conventional sealants.

Resin infiltrants represent a promising alternative due to their ability to penetrate and occlude microporosities within lesions, blocking diffusion pathways and contributing to the arrest of caries progression [9,10]. In recent years, the use of resin infiltrants has grown mainly due to their current indications—non-cavitated incipient lesions on smooth or proximal surfaces and, more recently, the esthetic correction of enamel defects—which has increased their clinical popularity [11]. In this context, expanding their application to occlusal surfaces would be particularly appealing, as it would make the material more versatile. However, the commercially available infiltrant is not yet indicated for occlusal use, possibly due to limitations related to its composition and mechanical properties. This lack of specific indication underscores the importance of investigating whether infiltration may also be effective on occlusal surfaces.

The aim of this systematic review was to evaluate the potential efficacy of resin infiltrants in non-cavitated occlusal carious lesions in primary and permanent teeth, with the goal of providing evidence to support the selection of less invasive yet potentially effective clinical treatments and clarifying the limitations associated with the use of resin infiltrants. The tested hypothesis was that resin infiltrants may exhibit clinical efficacy comparable to or potentially superior to that of other restorative materials for arresting the progression of carious lesions on occlusal surfaces.

## 2. Materials and Methods

### 2.1. Protocol and Registration

This systematic review followed the PRISMA (Preferred Reporting Items for Systematic Reviews and Meta-Analyses) checklist (Supplementary Material Table S1) [12], and its protocol was registered in the International Prospective Register of Systematic Reviews (PROSPERO) under number CRD42024606797.

### 2.2. Eligibility Criteria

The guiding question of this research was as follows: “Does the use of resin infiltrant in non-cavitated occlusal carious lesions suggest a potential method for controlling carious lesions in primary and permanent teeth?” The inclusion criteria for this review were based on the Population–Intervention–Comparison–Outcomes–Study Design (PICOS) strategy: (P)opulation—Non-cavitated occlusal carious lesions in primary or permanent teeth, defined as ICDAS scores 0–4: (0) sound tooth surface; (1): visible opacity or discoloration of the enamel, with no surface breakdown; (2): distinct visual change in the enamel, with no surface breakdown; (3): localized enamel breakdown due to caries, but without visible dentin involvement; and (4): underlying dentin shadow, indicating more advanced demineralization, but without cavitation; or equivalent diagnostic criteria adopted in vivo or in extracted teeth (ex vivo/in vitro). (I)ntervention—resin infiltrant applied to non-cavitated occlusal carious lesions. (C)omparison—resin infiltrant compared with placebo or a different material/technique. (O)utcomes—rate of progression of non-cavitated occlusal carious lesions. (S)tudy design—clinical, in vitro, and ex vivo studies.

The inclusion criteria adopted were: (1) clinical studies (randomized and non-randomized clinical trials); (2) sample settings involving patients or in vitro studies with specimens; (3) studies comparing resin infiltrant applied to non-cavitated occlusal carious lesions with placebo or a different technique; and (4) studies evaluating the progression rate of non-cavitated carious lesions. The exclusion criteria were: (1) clinical studies such as case reports or case series, retrospective and prospective studies; (2) studies evaluating the application of resin infiltrant on cavitated occlusal carious lesions; (3) unpublished information in the scientific literature; and (4) studies with full text unavailable

### 2.3. Data Sources and Search Strategy

To identify articles that evaluated the potential effectiveness of resin infiltrants applied to non-cavitated occlusal carious lesions, a search without geographic or language restrictions was conducted by two researchers (S.B.M. and J.A.M.d.S.) in the electronic databases PubMed/MEDLINE, Embase, Cochrane Library, Web of Science, Scopus and https://www.clinicaltrials.gov (non-peer-reviewed literature) in December 2025. This search used a combination of specific terms and keywords grouped using the Boolean operators AND and OR. Additionally, the reference lists of the included articles were manually screened to identify any further eligible studies. The search strategy applied for each database is described in Table 1.

### 2.4. Study Selection

All studies were imported into the Rayyan software (Rayyan Free version, Qatar Computing Research Institute, Ar-Rayyan, Qatar) [13]. After removing duplicate articles, the titles and abstracts were screened by two independent reviewers (S.B.M. and J.A.M.d.S.) to determine whether the studies met the predefined criteria. Based on the eligibility criteria, full-text reading was performed, and a third reviewer (R.B.E.L.) evaluated the inclusion and selection processes carried out by the other two reviewers. Any disagreements that arose during the independent screening were resolved through discussion. The article selection process was presented in a flowchart. Mendeley software 2.141.0 (Elsevier, Mendeley) was used as the reference manager. The Kappa Score [14] was used to assess the level of agreement between the reviewers regarding study inclusion. Eligible articles were read, and their data were carefully extracted.

### 2.5. Data Extraction

Data from the articles were manually extracted by a single reviewer (J.A.M.d.S.) and reviewed by a second reviewer (S.B.M.). Any discrepancies between reviewers were resolved through discussion with a third reviewer (R.B.E.L.). The extracted data were organized in a standardized Excel spreadsheet (Microsoft, Redmond, WA, USA). The variables collected from the studies included: author, year of publication, study design, sample size (age range of participants and number of teeth), type of sample (patients or evaluation unit), initial lesion depth, resin infiltrant, intervention, comparison, follow-up period, method used to assess caries progression rate, caries progression rate, main results, and adverse events. Tables were prepared to present the characteristics of interest.

### 2.6. Risk-of-Bias Assessment

The risk of bias for all eligible studies was assessed by two independent reviewers (S.B.M. and J.A.M.d.S.) who were calibrated prior to the assessment, using two tools. Any disagreements were resolved through discussion or, when necessary, by a third reviewer (R.B.E.L.). For RCTs, the risk of bias was evaluated using the Cochrane Risk of Bias Tool for RCTs (RoB 2) [15], in which the assessment criteria were divided into five domains: (1) bias arising from the randomization process; (2) bias due to deviations from intended interventions; (3) bias due to missing outcome data; (4) bias in the measurement of the outcome; and (5) bias in the selection of the reported result. The domains were classified as “no information,” “low,” “unclear,” or “high” risk of bias for each study. For a study to be considered at low risk of bias, all key domains needed to be rated as “low.” If one domain was rated as “unclear,” the study was considered to raise some concerns. If two domains were rated as “unclear” or at least one as “high,” the study was considered to have a high risk of bias.

For in vitro studies, the risk of bias was assessed using seven parameters for evaluating study quality, adapted from a systematic review of in vitro studies [16]: (1) randomization of teeth; (2) teeth free of caries; (3) standardization of enamel or dentin surface samples; (4) sample size; (5) examiner blinding; (6) sample size calculation; and (7) reporting of complete outcome data. If the authors reported the parameter, the study received an “S” (yes) for that specific item. If the information was not found, the study received an “N” (no). Studies reporting one to three “S” items were classified as having a high risk of bias, four or five as medium risk, and six or seven as low risk. All domains in the risk-of-bias tool were given equal weight, ensuring a comprehensive evaluation of each study’s methodological quality. While this tool was adapted, it has demonstrated good reliability and validity for evaluating in vitro studies, as shown in previous systematic reviews. Its inter-rater reliability has been consistently high, and it remains a valid instrument for assessing the methodological quality of in vitro studies.

### 2.7. Assessment of Certainty of Evidence

The certainty of the evidence of the included studies was evaluated using the Grading of Recommendations, Assessment, Development, and Evaluation (GRADE) tool [17], available at http://www.gradeworkinggroup.org/. This tool considers the study design and assesses factors such as risk of bias, imprecision, inconsistency, indirectness of evidence, and publication bias to determine the overall quality of the evidence. Each aspect was rated as “no limitation,” “serious limitations,” or “very serious limitations,” allowing the evidence to be classified as high, moderate, low, or very low quality. When evidence is rated as lower quality, this indicates that the estimated effect may differ substantially from the true effect. Although GRADE is primarily used for clinical studies, its application in this review was adapted for in vitro research. Laboratory studies often involve controlled conditions that differ from clinical environments, introducing indirectness. To address this, the GRADE assessment for laboratory outcomes (e.g., penetration depth, microleakage, and fluorescence) was downgraded due to the limited applicability of these findings to real-world clinical settings. While valuable for understanding the mechanisms of resin infiltrants, the laboratory results must be interpreted with caution.

### 2.8. Strategy for Data Synthesis

A meta-analysis was not possible due to the heterogeneity of the protocols used in the included studies. This heterogeneity involved substantial variations in the application methods of the resin infiltrant, the types of comparators used, and the different evaluation methods. Additionally, most studies involved in vitro and ex vivo models, whose results may not reflect actual clinical conditions, making it difficult to combine data in a meaningful and robust way. As an alternative, a narrative synthesis was performed, allowing qualitative analysis of the findings and interpretation of the available data despite methodological limitations and lack of uniformity in the assessed outcomes.

## 3. Results

### 3.1. Study Selection

**Figure 1:**
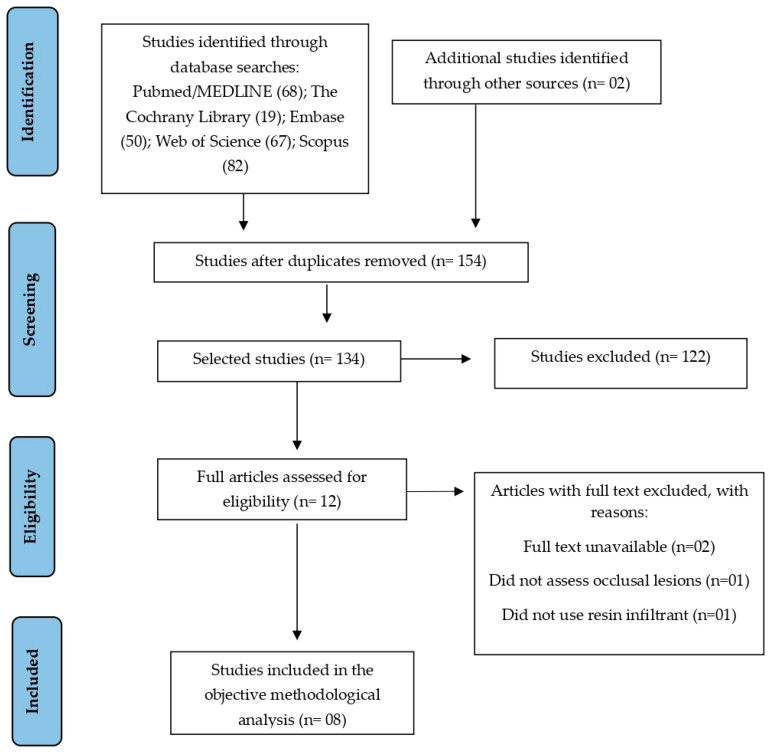
Flowchart of articles included in the systematic review.

A comprehensive electronic search across five databases—PubMed/MEDLINE (n = 68), The Cochrane Library (n = 19), Embase (n = 50), Web of Science (n = 67), and Scopus (n = 82)—resulted in a total of 286 records, plus 2 additional records from other sources. No clinical trial records were identified in the non-peer-reviewed literature (https://www.clinicaltrials.com). After removing duplicates, 134 unique records remained. Title and abstract screening were then performed, leading to the exclusion of 122 studies that did not meet the inclusion criteria. A total of 12 full-text articles were assessed for eligibility, of which 8 studies met the predefined criteria and were included in the final qualitative and methodological synthesis. The selection process is summarized in the PRISMA flowchart (Figure 1).

The results of the inter-examiner agreement test showed an “almost perfect agreement” between the examiners in the article selection phase. The indices of the databases were PubMed/Medline (0.8), Embase (1.0), The Cochrane Library (1.0), Web of Science (1.0), and Scopus (0.8). The overall kappa for title/abstract screening, combining the results from all databases, was found to be 0.92.

### 3.2. Characteristics of the Studies

The characteristics of the studies are observed in Table 2.

This review included eight studies, comprising two RCTs with a split-mouth design and six in vitro/ex vivo investigations. In vitro and ex vivo studies were included in this review primarily to investigate the underlying mechanisms, such as the penetration and sealing capacity of resin infiltrants. However, these studies do not provide direct evidence of clinical efficacy and should be interpreted as preliminary findings that help inform clinical research.

The RCTs involved pediatric and young adult populations, with sample sizes ranging from 23 to 47 participants, and evaluated primary and permanent molars presenting initial carious lesions classified as ICDAS 1 to 4.

Most in vitro studies used extracted premolars and molars with artificially or naturally developed enamel lesions, with depths up to ~1232 μm, including cases in which demineralization extended to the enamel–dentin junction (EDJ) or to outer dentin.

All studies evaluated the use of the ICON^®^ resin infiltrant (DMG, Germany) as the main intervention. Most protocols included conditioning with 15% hydrochloric acid (HCl), followed by ethanol drying and resin application. Several studies explored modifications to this protocol, such as additional abrasion, brushing, extended application time, or combining infiltration with flowable composite resin. Comparators varied across studies and included fluoride varnish, conventional sealants, flowable resin applied alone, or no treatment.

Follow-up periods in the clinical trials ranged from 8 to 34 months and up to 3 years, while all laboratory studies assessed results immediately after treatment. Evaluation methods included bitewing radiographs, DIAGNOdent, stereomicroscopy, QLF (Quantitative Light-Induced Fluorescence), polarized light microscopy, and confocal laser scanning microscopy (CLSM).

### 3.3. Caries Progression Rate of RCTs

In this review, caries progression was defined as any increase in the severity of a carious lesion, including changes in visual appearance, radiographic findings, or increased lesion depth, such as the development of cavitation. The methods used to assess caries progression varied across the studies included in the review, and these methods included: radiographs, clinical examination, laser fluorescence (DIAGNOdent), and quantitative light-induced fluorescence (QLF). The studies varied in the specific methods used, and the assessment of caries progression was made based on the available diagnostic tools.

The clinical studies reported lower rates of caries progression in groups treated with resin infiltrant compared with control groups—fluoride alone [18] and conventional resin sealant [9]. In one of the trials, the difference was statistically significant in favor of infiltration. No adverse events were reported in the clinical studies (Table 3).

In the study by Bakhshandeh and Ekstrand [18], the group treated with resin infiltrant combined with fluoride varnish (I+F) showed the lowest caries progression rate (15%), significantly lower than the fluoride-only group (F) (36%; *p* = 0.021). The sealant + fluoride varnish group (S+F) showed a progression rate of 19%, with no statistically significant difference when compared to group F (*p* = 0.096) or group I+F (*p* = 0.774).

In the study by Anauate-Netto et al. [9], both the resin infiltrant group and the conventional sealant group showed similarly low progression rates (2.4% and 2.5%, respectively), with no statistically significant difference between them.

### 3.4. Penetration, Microleakage, and Fluorescence Outcomes of Laboratory Studies

Several in vitro and ex vivo studies evaluated the performance of resin infiltrants through measurements of penetration depth, microleakage, and fluorescence loss. Overall, the findings indicate that optimized pretreatment protocols—especially those including brushing or abrasion—promote greater resin penetration, although deeper lesions or those with thicker surface barriers continue to present challenges to infiltration (Table 4).

In vitro and ex vivo studies provide crucial mechanistic insights into the penetration depth, microleakage, and fluorescence changes associated with resin infiltrants. These laboratory studies are valuable for understanding the material’s performance under controlled conditions. However, it is important to note that these laboratory performance outcomes do not directly correlate with clinical efficacy in terms of lesion progression/arrest. The clinical trials included in this review provide preliminary evidence suggesting that resin infiltrants may reduce caries progression; however, the evidence is limited, and further well-conducted long-term trials are needed to confirm their clinical effectiveness.

Paris et al. [19] observed the highest percentage of maximum penetration (PPmax) using ICON^®^ (41%), compared to 11% when phosphoric acid alone was used and 5% with conventional sealants. Similar results were reported by Lausch et al. [20], who found significantly higher infiltration when ICON^®^ was applied after brushing abrasion for 120 s (64%) or 30 s (61%), whereas the standard HCl-based protocol resulted in a lower infiltration rate (23%). Supporting these findings, Meyer-Lueckel et al. [24] confirmed that combining abrasion with HCl conditioning (H30BA—15% HCl for 30 s + Abrasion + Brushing) promoted nearly complete infiltration, outperforming both the manufacturer’s recommended protocol and phosphoric acid-based techniques.

Regarding microleakage, Kielbassa et al. [21] reported better performance for the ICON + flowable resin combination, which showed a lower frequency of leakage (1/20) and fewer air bubbles (2/20) compared with flowable resin alone (5/20 and 17/20, respectively). The authors also observed greater penetration in premolars (57.9%) than in molars (35.3%), indicating that dental morphology influences infiltrant performance.

Concerning fluorescence outcomes, Silva FG et al. [22] recorded a significant reduction in fluorescence loss (ΔF and ΔQ) after infiltration, whereas untreated controls showed no changes. In another study, Silva VB et al. [23] demonstrated that the infiltrant + flowable resin combination provided effective immediate sealing in 80% of samples, compared with only 30% sealing when flowable resin was used alone.

### 3.5. Risk of Bias in the Studies

The risk of bias among the included in vitro and ex vivo studies was generally moderate to low (Table 5).

All six studies reported adequate randomization of teeth and the use of caries-free teeth, contributing to baseline sample comparability. Additionally, the standardization of enamel and dentin surfaces was consistently reported. However, only two studies provided evidence of examiner blinding during outcome assessment [21,22].

Although all studies reported the number of specimens analyzed, the absence of a priori sample size calculation in most investigations may have increased the risk of imprecision. Sample size calculation was explicitly reported only in the study by Silva FG et al. [22], indicating more rigorous methodological planning. Nevertheless, all studies provided complete outcome data, mitigating concerns regarding attrition bias.

As a result, two studies [21,22] were classified as having low risk of bias, while the remaining studies were categorized as having moderate risk, mainly due to a lack of reporting on examiner blinding and sample size calculation.

**Figure 2:**
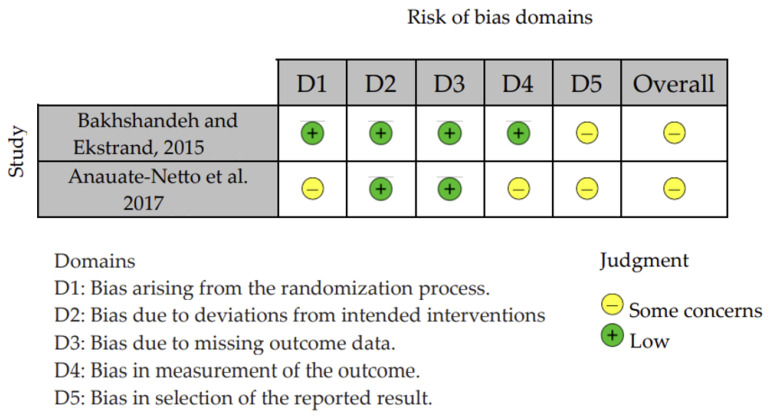
Risk-of-bias analysis for RCTs (RoB2) [9,18].

The risk of bias of the two RCTs included was assessed using the Cochrane Risk of Bias 2.0 (RoB 2) tool. The study by Bakhshandeh and Ekstrand [18] was rated as having a low risk of bias in the domains related to the randomization process (D1), deviations from intended interventions (D2), and missing outcome data (D3). However, the study presented some concerns in outcome measurement (D4) and reported result selection (D5), leading to an overall judgment of “some concerns” (Figure 2).

Similarly, Anauate-Netto et al.’s study [9] was considered to have low risk in D2 and D3 but showed some concerns regarding randomization (D1), outcome measurement (D4), and reporting bias (D5). As a result, this trial was also judged as having an overall risk of bias of “some concerns.”

Although both studies maintained low risk in key methodological aspects, such as intervention administration and outcome completeness, the presence of concerns in measurement and reporting domains limits the certainty of their findings.

### 3.6. Certainty of Evidence

The GRADE assessment of evidence quality showed that the certainty of the evidence ranged from moderate to low across the outcomes analyzed. Evidence was considered of moderate quality for the outcome of infiltrant penetration, based on six studies (five in vitro and one randomized clinical trial), with one level of downgrading due to serious risk of bias. For the other outcomes—microleakage/sealing, fluorescence (ΔF), and lesion progression—the evidence quality was rated as low, mainly due to risk of bias and imprecision. This classification reflects the predominance of laboratory studies and the limited number of well-conducted clinical trials, which restrict the robustness of the conclusions (Table 6).

## 4. Discussion

Globally, dental caries remains a major oral health problem, and non-cavitated occlusal lesions continue to pose a challenge for minimally invasive dentistry [25,26]. In addition to their high prevalence, these lesions present greater diagnostic complexity in their early stages and a high risk of progression [9]. In this context, resin infiltrants such as ICON^®^ have emerged as a conservative alternative capable of halting caries progression while preserving as much healthy tooth structure as possible [27,28]. This systematic review evaluated the potential effectiveness of resin infiltrants in non-cavitated occlusal caries lesions, based on both clinical outcomes—such as caries progression measured by radiographic examinations and visual criteria—and laboratory outcomes, including penetration depth, microleakage, and fluorescence changes.

The hypothesis of the study was that resin infiltrants may exhibit similar or better efficacy compared to traditional approaches used to manage non-cavitated occlusal caries lesions. Based on the results obtained, this hypothesis can be considered partially supported, since infiltration showed potentially better performance compared to fluoride varnish and comparable performance to conventional sealants.

The clinical trials included demonstrated that resin infiltration may reduce caries progression when compared with the isolated use of fluoride varnish [18], suggesting better performance under these conditions. On the other hand, when compared with conventional sealants, resin infiltration showed similar results, without statistically significant differences in lesion progression [9]. These findings suggest that resin infiltrants may offer an effective alternative to traditional treatments, either by potentially outperforming fluoride varnish or by providing similar efficacy to fissure sealants.

Resin infiltrants have the ability to penetrate the subsurface porosities of early lesions, increasing the microhardness of demineralized enamel due to their formulation based on low-viscosity resins [21]. Their mechanism of action is based on capillary forces, and infiltration efficacy is directly influenced by capillary radius and pore volume, as described by the Washburn equation [29]

For optimal penetration, prior acid conditioning is required to expose the porous structure and allow resin uptake [23,30]. This is because the highly mineralized surface layer of the lesion acts as a barrier to infiltration [30]. Thus, resin infiltrants provide mechanical stabilization of the tooth structure and help stop caries progression, in accordance with the principles of preventive and minimally invasive dentistry [20,31]. The studies included in this review [9,18,19,20,22,23,24] suggest valuable insights into the potential effectiveness of these materials across different clinical and laboratory settings.

The clinical trials [9,18] reinforce the potential of resin infiltrants for managing non-cavitated occlusal lesions, though their efficacy varies depending on the comparator material. The absence of a statistical difference between infiltrants and sealants may be explained by the fact that both create a resin-based mechanical barrier capable of blocking the diffusion of acids and bacterial byproducts, interrupting lesion progression [19,21]. Nevertheless, the materials have distinct characteristics. Infiltrants show greater penetration depth into enamel [19,21], whereas sealants remain more superficial and contain filler particles [10]. These structural differences may influence clinical performance and justify further studies to clarify their long-term impact on durability and treatment effectiveness.

Anauate-Netto et al. [9] also found no statistically significant differences between infiltrants and conventional sealants, as both presented low progression rates. Although these findings suggest that both materials may be effective minimally invasive options in the short term, the choice between them may depend on factors that have not yet been thoroughly assessed clinically. Elements such as the stability of the formed layer, the mechanical behavior of the treated enamel, and the implications of different depths of interaction with the substrate may influence long-term performance. Therefore, although current evidence does not support recommending one material over the other categorically, these differences suggest potential specific advantages, reinforcing the need for further investigations evaluating these variables more comprehensively.

Another outcome evaluated was resin penetration depth. Laboratory studies consistently suggested better performance when extended preparation protocols were used compared with the standard manufacturer-recommended protocol. Paris et al. [19] demonstrated that infiltrants achieved greater penetration depth than conventional sealants, whereas soft-etching with phosphoric acid (H_3_PO_4_) resulted in markedly inferior penetration. Soft-etching only partially removes the lesion surface layer, unlike HCl conditioning, which enables more effective removal and facilitates resin infiltration. This highlights that application protocol selection directly affects infiltration efficacy. Similarly, Lausch et al. [20] observed significantly greater infiltration when abrasion with brushes was added to HCl conditioning, emphasizing the importance of combined conditioning strategies.

Consistent with these results, Meyer-Lueckel et al. [24] confirmed that near-complete penetration was achieved only with the HCl-plus-abrasive protocol, showing that more intense removal of the lesion surface layer is a key factor for treatment success. Kielbassa et al. [21] further demonstrated that premolars exhibited greater penetration compared with molars, suggesting that tooth morphology also influences infiltration effectiveness. These findings confirm that optimal penetration depends not only on the properties of the infiltrant but also on surface characteristics and preparation protocol. However, it is important to consider that overly long protocols may not be clinically practical, as they increase chair time and the risk of additional tooth structure removal. Thus, balancing the maximal efficacy observed in laboratory settings with everyday clinical applicability remains a challenge.

Regarding microleakage, the studies by Kielbassa et al. [21] and Silva VB et al. [23] showed that the combined use of infiltrant with flowable resin provided better sealing and fewer air bubbles than the use of flowable resin alone. This favorable interaction suggests that infiltration may enhance the performance of other restorative materials, serving as a complementary step in sealing protocols.

With respect to fluorescence, Silva VB et al. [23] found a significant reduction in fluorescence loss after infiltration, reflecting the optical change resulting from the filling of lesion porosities with resin—a key principle underlying the infiltrant’s ability to stabilize lesions and inhibit caries progression. Although such laboratory evidence is limited to controlled conditions and short-term analyses, it reinforces the ability of infiltrants to create effective barriers against lesion advancement.

Compared with other management strategies, resin infiltrants suggested similar or better performance depending on the clinical context. Bakhshandeh and Ekstrand [18] found an advantage of combining infiltrant with fluoride varnish over fluoride varnish alone. Conversely, Anauate-Netto et al. [9] observed that infiltrants performed comparably to conventional sealants, with both presenting low caries progression rates. This similarity suggests that infiltration can be considered a minimally invasive alternative to sealants, especially in cases where mechanical retention may be limited. In addition, optimized infiltration protocols demonstrated greater penetration and sealing ability than conventional sealing techniques in laboratory studies [19,20,21,22,23,24], which may explain the clinical equivalence observed. However, it remains unclear whether this laboratory advantage may translate into additional long-term clinical benefits when compared with well-established techniques such as sealants, which have a longer clinical history and stronger evidence base.

Overall, the promising results of this systematic review reveal consistency between clinical and laboratory findings regarding the potential of resin infiltrants to halt the progression of non-cavitated occlusal lesions. The findings from RCTs demonstrate a reduction and arrest in caries progression, while laboratory studies provide valuable insights into the mechanisms of action of resin infiltrants, such as penetration depth, microleakage, and fluorescence changes. While these laboratory findings help understand the material’s properties, it is important to emphasize that they cannot, by themselves, support claims about clinical efficacy, which must be evaluated based on direct clinical outcomes.

However, several methodological limitations restrict the strength of the conclusions. The limited number of clinical trials with short follow-up periods (8–34 months and 3 years) restricts our ability to make concrete conclusions about the long-term clinical effectiveness and durability of resin infiltrants. While resin infiltrants show promise, there is a significant need for additional well-designed long-term RCTs to assess their sustained clinical outcomes. Furthermore, although laboratory studies are valuable for elucidating mechanisms and physical–chemical behavior, they do not fully reproduce clinical conditions such as salivary variability, dietary habits, and adherence to preventive measures, reducing the generalizability of results.

Laboratory studies provide valuable insights into the mechanisms of resin infiltrants, including penetration depth, microleakage, and fluorescence changes, but these surrogate outcomes have limitations when applied to clinical practice. Although they help explain the material’s behavior under controlled conditions, they do not directly predict clinical efficacy. Factors like caries progression, restoration longevity, and patient satisfaction depend on more complex conditions that laboratory models cannot fully replicate.

Despite laboratory study success with resin infiltrants, the low-to-moderate evidence quality (GRADE) indicates that extended clinical trials are essential for proper evaluation of clinical use performance. These trials should focus on direct clinical outcomes, such as caries progression, marginal integrity, and restoration survival, to provide a more comprehensive understanding of the treatment’s true effectiveness.

Although resin infiltrants show promising results in managing non-cavitated occlusal lesions, the certainty of the evidence is classified as low to moderate based on the GRADE assessment. The conclusions drawn from the available studies are limited by methodological concerns, small sample sizes, and short follow-up periods. Given these limitations, further rigorous clinical studies are necessary to confirm the clinical effectiveness and durability of resin infiltrants. Due to the limited number of included studies, especially clinical trials, a formal assessment of publication bias was not feasible. The potential for publication bias exists, as studies with positive or significant findings may be more likely to be published. This limitation should be considered when interpreting the findings of this review.

The inability to perform a meta-analysis due to methodological differences between studies means that conclusions are based on the results of individual studies, which may not be reproducible. The heterogeneity between studies, including variations in HCl concentration, treatment duration, and methods of measuring penetration, as well as differences in control groups, should be considered when interpreting the efficacy of resin infiltrants. These factors could impact the outcomes and contribute to variations in the clinical effectiveness observed across studies.

The inclusion of ICDAS 4 lesions, which represent more advanced demineralization without cavitation, may influence the interpretation of treatment outcomes. Although ICDAS 4 lesions do not exhibit cavitation, they are more advanced than ICDAS 1-3 lesions, with an underlying dentin shadow indicating significant enamel demineralization. This could affect the clinical effectiveness of resin infiltration, as more advanced lesions may be less responsive to treatment or may require different therapeutic approaches compared to less advanced lesions. The inclusion of ICDAS 4 lesions in this review reflects the variability in lesion progression and diagnostic criteria across studies. However, the impact of deeper lesions on treatment effectiveness should be considered, as deeper lesions may require more intensive interventions or may show different treatment outcomes than earlier-stage non-cavitated lesions.

Therefore, although resin infiltrants still require long-term clinical validation, current evidence positions them as a promising alternative within minimally invasive dentistry, expanding the range of conservative therapeutic options available in clinical practice. Future research should prioritize well-designed clinical trials with larger samples, extended follow-up, and standardized protocols to assess not only the effectiveness of infiltrants in preventing the progression of occlusal lesions but also their impact on broader clinical parameters such as sealing longevity, patient satisfaction, and cost-effectiveness compared with other minimally invasive techniques.

## 5. Conclusions

Based on the available clinical trials, resin infiltrants show promising results in managing non-cavitated occlusal lesions, with evidence suggesting reduced caries progression compared to fluoride varnish and comparable efficacy to conventional sealants. These conclusions are primarily supported by clinical data, which demonstrate the real-world effectiveness of resin infiltrants in halting caries progression. Laboratory studies, including those evaluating penetration depth, microleakage, and fluorescence changes, provide valuable insights into the mechanisms underlying these clinical effects but should be considered as supportive evidence to explain the observed clinical outcomes. Given the limitations of the available evidence, including the short follow-up periods and small sample sizes in the clinical trials, further well-designed, long-term randomized controlled trials are necessary to confirm the sustained clinical effectiveness and durability of resin infiltrants.

## Figures and Tables

**Table 1:** Electronic search strategies.

Database	Search Strategy
PubMed	(resin infiltration OR caries infiltration OR infiltrate resin OR infiltration OR infiltrant) AND (occlusal caries OR fissure caries OR fissure caries lesion OR non-cavitated carious lesions OR non-cavitated fissures OR occlusal surfaces OR early occlusal caries) AND (Penetration ability OR caries progression)
Embase	(‘resin infiltration’/exp OR ‘resin infiltration’ OR ((‘resin’/exp OR resin) AND (‘infiltration’/exp OR infiltration)) OR ‘caries infiltration’ OR ((‘caries’/exp OR caries) AND (‘infiltration’/exp OR infiltration)) OR ‘infiltrate resin’ OR ((‘infiltrate’/exp OR infiltrate) AND (‘resin’/exp OR resin)) OR ‘infiltration’/exp OR infiltration OR infiltrant) AND (‘occlusal caries’/exp OR ‘occlusal caries’ OR ((‘occlusal’/exp OR occlusal) AND (‘caries’/exp OR caries)) OR ‘fissure caries’ OR ((‘fissure’/exp OR fissure) AND (‘caries’/exp OR caries)) OR ‘fissure caries lesion’ OR ((‘fissure’/exp OR fissure) AND (‘caries’/exp OR caries) AND (‘lesion’/exp OR lesion)) OR ‘non-cavitated carious lesions’ OR (‘non cavitated’ AND carious AND lesions) OR ‘non-cavitated fissures’ OR (‘non cavitated’ AND fissures) OR ‘occlusal surfaces’ OR ((‘occlusal’/exp OR occlusal) AND surfaces) OR ‘early occlusal caries’ OR (early AND (‘occlusal’/exp OR occlusal) AND (‘caries’/exp OR caries))) AND (‘penetration ability’ OR ((‘penetration’/exp OR penetration) AND (‘ability’/exp OR ability)) OR ‘caries progression’ OR ((‘caries’/exp OR caries) AND (‘progression’/exp OR progression)))
Web of Science	(TS=(Penetration ability OR caries progression)) AND TS=((resin infiltration OR caries infiltration OR infiltrate resin OR infiltration OR infiltrant) AND (occlusal caries OR fissure caries OR fissure caries lesion OR non-cavitated carious lesions OR non-cavitated fissures OR occlusal surfaces OR early occlusal caries) AND (Penetration ability OR caries progression))
The Cochrane Library	(resin infiltration OR caries infiltration OR infiltrate resin OR infiltration OR infiltrant) AND (occlusal caries OR fissure caries OR fissure caries lesion OR non-cavitated carious lesions OR non-cavitated fissures OR occlusal surfaces OR early occlusal caries) AND (Penetration ability OR caries progression)
Scopus	(resin AND infiltration OR caries AND infiltration OR infiltrate AND resin OR infiltration OR infiltrant) AND (occlusal AND caries OR fissure AND caries OR fissure AND caries AND lesion OR non-cavitated AND carious AND lesions OR non-cavitated AND fissures OR occlusal AND surfaces OR early AND occlusal AND caries) AND (penetration AND ability OR caries AND progression)

**Table 2:** Characteristics of included studies.

Author, Year	Design of Study	Lesion Type	n	Sample	Initial Depth of Lesion	Resin Infiltrant	Intervention	Comparison	Follow-Up Period	Method of Assessing Caries Progression Rate	Caries Progression Rate	Main Results	Adverse Events
Bakhshandeh and Ekstrand, 2015 [18]	RCT, split-mouth.	Natural	47 patients (150 teeth)	Deciduous molars (ICDAS 1, 2, and 4). Age range: 5 to 8 years.	84%—Enamel–dentin junction (EDJ) or outer third of dentin.	ICON^®^ (I) (DMG Chemisch-Pharmazeutische Fabrik GmbH, Hamburg, Germany).	15% HCl + 95% ethanol + ICON Infiltrant + Duraphat.	Delton sealant (S) + Duraphat fluoride varnish (F).	8 to 34 months.	Interproximal radiographs.	I+F (15%), S+F (19%), and F (36%); a significant difference was found between I+F and F (*p* = 0.021).	I+F was significantly higher than F (*p* = 0.021).	No adverse events reported.
Paris et al. 2014 [19]	In vitro	Artificial		Premolars and molars (ICDAS 0, 1, and 2).	Até 1192 (805–1512) μm	ICON (DMG, Hamburg, Germany).	ICON Infiltrant with 15% HCl or 37% H_3_PO_4_.	Duraphat fluoride varnish (F) alone.	Immediate evaluation.	Laser scanning confocal microscopy (CLSM).	Not applicable.	Greater penetration with HCl + Icon (PPmax 41%) compared to H_3_PO_4_ (11%) and sealant (5%).	Limited penetration due to fissures, biofilm, and air bubbles.
Lausch et al. 2015 [20]	In vitro	Artificial	123 teeth.	Premolars and molars (ICDAS 2).	~1232 μm	Icon (DMG, Hamburg, Germany).	ICON after 15% HCl, with variations: abrasives, type of brush, and application time.	Sealing with Helioseal.	Immediate evaluation.	Confocal fluorescence microscopy.	Not applicable.	Microabrasion with a stiff brush + HCl promoted greater penetration (almost complete).	Limited penetration due to morphology, biofilm, and air bubbles.
Anauate-Netto et al. 2017 [9]	RCT split-mouth.	Natural	23 patients (86 teeth)	Permanent molars (ICDAS 1 to 3). Age range: 8 to 24 years.	Between the EDJ and the middle third of dentin	Icon^®^ (DMG, Germany).	ICON, 15% HCl, ethanol, and infiltrant for 2 min.	Conventional 15% HCl for 120 s + Icon for 180 s.	3 years.	Interproximal radiographs, DIAGNOdent, explorer probe, and scanning electron microscopy (SEM).	Infiltrant (2.4%) and sealant (2.5%); laser fluorescence: infiltrant (2.6%) and sealant (5.6%).	Similar efficacy; the infiltrant showed better marginal integrity and stability after 1 year.	Marginal integrity decreased after 1 year and stabilized in years 2 and 3.
Kielbassa et al. 2017 [21]	Ex vivo	Artificial	40 teeth.	Premolars and molars (ICDAS 2). Age range: not specified.	EDJ (detected by DIAGNOdent)	Icon^®^ (DMG, Hamburg, Germany).	ICON + sealing with G-ænial Flow (GC Europe).	Sealant (Alpha Seal).	Immediate evaluation.	Confocal microscopy (CLSM).	Not applicable.	Infiltration rate: 57.9% (premolars), 35.3% (molars); less microleakage and fewer bubbles in RI/CR than in CR.	No adverse events reported.
Silva FG et al. 2020 [22]	In vitro	Natural	60 teeth.	Permanent teeth (ICDAS 1, 2, and 3).	Mean lesion area: ICDAS 1 = 0.70 mm^2^; ICDAS 2 = 3.10 mm^2^; ICDAS 3 = 2.99 mm^2^.	Icon Infiltrant^®^ (DMG, Hamburg, Germany).	ICON with 15% HCl + ethanol + two layers of resin.	Sealing with G-ænial Flow, without infiltration.	Immediate evaluation.	QLF for lesion area, ΔF and ΔQ, confirmed by polarized light microscopy.	Not applicable.	Reduction in post-treatment values; greater resin penetration in ICDAS 2 and 3 lesions.	Incomplete infiltration in ICDAS 3 lesions; lower penetration in ICDAS 1 lesions.
Silva VB et al. 2020 [23]	In vitro.	Artificial	20 teeth.	Third molars.	Micro-CT with E1, E2, D1, and D2. Surface demineralization to the involvement of outer dentin.	Icon (DMG America, Ridgefield Park, NJ, USA).	Resin infiltrant + flowable resin.	Pre- and post-treatment with resin infiltration in ICDAS 1, 2, and 3 lesions; no control group.	Immediate evaluation.	Microleakage test using 3% methylene blue, analyzed under a stereomicroscope (25×).	Not applicable.	Infiltrant + flowable resin—effective immediate sealing of 80%; flowable resin alone—immediate sealing of 30%.	No adverse events reported.
Meyer-Lueckel et al. 2022 [24]	In vitro	Artificial	60 teeth	Molars (ICDAS 2).	Depth: 537 μm (range: 274–876 μm). Thickness of the non-infiltrated surface layer: 33 μm (23–51 μm).	Icon (DMG, Germany)	Pretreatment techniques: 15% HCl for 120 s (H120); 15% HCl with brushing (H30B); 15% HCl with abrasive and brushing (H30BA); 37% H_3_PO_4_ for 120 s (P120); H_3_PO_4_ with abrasive and brushing (P120BA). After pretreatment, standard infiltration with Icon^®^ (DMG, Germany) was performed for 180 s.	Flowable resin.	Immediate evaluation.	Measurement of SL, LD, and PD by CLSM. Laser scanning confocal microscopy with fluorescence.	Not applicable.	The H30BA group (HCl + abrasive + brushing) showed: greater removal of the superficial layer; greater infiltrant penetration (almost complete). Groups with phosphoric acid (P120 and P120BA): less erosion of the superficial layer; lower resin penetration. Groups with HCl (without brushing/abrasive) showed intermediate results.	Infiltration limitations in the phosphoric acid groups.

**Table 3:** Caries progression rate of RCTs.

Author, Year	Groups	n (Lesions)	Lesions with Progression	Caries Progression Rate	Statistical Difference
Bakhshandeh and Ekstrand, 2015 [18]	Resin infiltrant + fluoride varnish	47	7	15%	(*p* = 0.021) vs. F (*p* = 0.774) vs. S+F
	Sealant + fluoride varnish (S+F)	47	9	19%	(*p* = 0.096) vs. F
	Fluoride varnish only (F)	47	17	36%	-
Anauate-Netto et al. [9]	Resin infiltrant	42	1	2.4%	No significant difference
	Conventional sealant	40	1	2.5%	No significant difference

**Table 4:** Microleakage, fluorescence, and penetration of laboratory studies.

Author, Year	Outcome	Groups	n	Result	Unit/Indicator
Paris et al. 2014 [19]	Penetration (PPmax)	Icon (ICDAS 2)	9	41% (30–78%) 5% (0–9%) 11% (0–21%)	Maximum penetration %
		Selant (ICDAS 2)	10		
		Soft-Etch (H_3_PO_4_) (ICDAS 2)	9		
Lausch et al. 2015 [20]	Penetration	HCl + abrasive (120 s brushing)	20	64% 61% 23%	% of completely infiltrated fissures
		HCl + abrasive (30 s brushing)	20		
		Standard HCl (120 s)	20		
Kielbassa et al. 2017 [21]	Microleakage	Icon + flowable resin	20	1/20; Bubbles 2/20 5/20; Bubbles 17/20	Absolute frequency
		Flowable resin alone	20		
	Penetration	Icon + flow (premolars)	10	57.9% ± 23.1% 35.3% ± 22.1%	% infiltrated area
		Icon + flow (molars)	10		
Silva FG et al. 2020 [22]	Fluorescence (ΔF, ΔQ)	Icon	60	Significant reduction in fluorescence loss (*p* < 0.05) Values remained unchanged	ΔF (% fluorescence)
		Control (no treatment)	60		
Silva VB et al. 2020 [23]	Microleakage	Icon + flow	20	Effective sealing in 80% of the samples (score 0 or 1) Effective sealing in 30% of the samples (score 0 or 1)	% effective sealing (microleakage score 0 or 1)
		flow alone	20		
Meyer-Lueckel et al. 2022 [24]	Penetration	HCl + abrasive (H30BA)	12	Almost complete penetration Less than H30BA—intermediate penetration Overall lower penetration	Qualitative + confocal Comparative Comparative Comparative
		Standard HCl (H120)	12		
		H_3_PO_4_ + abrasive (P120BA)	12		
		Standard H_3_PO_4_ (P120)	12		

**Table 5:** Risk-of-bias assessment.

Study	Teeth Randomization	Teeth Free of Caries	Standardization of Enamel/Dentine Surface Samples	Sample Size	Blinding of the Examiner	Sample Size Calculation	Complete Outcome Data	Risk of Bias
Paris et al. 2014 [19]	Yes	Yes	Yes	Yes	NR	NR	Yes	Moderate
Lausch et al. 2015 [20]	Yes	Yes	Yes	Yes	NR	NR	Yes	Moderate
Kielbassa et al. 2017 [21]	Yes	Yes	Yes	Yes	Yes	NR	Yes	Low
Silva FG et al. 2020 [22]	Yes	Yes	Yes	Yes	Yes	Yes	Yes	Low
Silva VB et al. 2020 [23]	Yes	Yes	Yes	Yes	NR	NR	Yes	Moderate
Meyer-Lueckel et al. 2022 [24]	Yes	Yes	Yes	Yes	NR	NR	Yes	Moderate

NR: Not reported.

**Table 6:** Quality of evidence—GRADE assessment.

Certainty Assessment									
Parameter	No of Studies	Study Design	Risk of Bias	Inconsistency	Indirectness	Imprecision	Other Considerations	Important	Certainty
Infiltrant penetration	6	5 in vitro, 1 RCT	Serious ^a^	Not serious ^c^	Not serious ^d^	Not serious ^e^	None	-	⊕⊕⊕⊝ Moderate
Microleakage/sealing	4	3 in vitro, 1 RCT	Serious ^a^	Not serious ^c^	Not serious^d^	Serious ^f^	None	-	⊕⊕⊝⊝ Low
Fluorescence (ΔF)	1	In vitro	Not serious ^b^	Not applicable	Not serious ^d^	Serious ^f^	None	-	⊕⊕⊝⊝ Low
Lesion progression (Clinical)	2	RCT	Serious ^a^	Not serious ^c^	Not serious ^d^	Serious ^f^	None	-	⊕⊕⊝⊝ Low

Classification of the quality of evidence according to the GRADE Working Group. ⊕⊕⊕⊕ High quality: It is very unlikely that further research will change our confidence in the estimate of effect. ⊕⊕⊕⊝ Moderate quality: Further research is likely to have an important impact on our confidence in the estimate of effect and may change the estimate. ⊕⊕⊝⊝ Low quality: Further research is very likely to have an important impact on our confidence in the estimate of effect and is likely to change the estimate. ⊕⊝⊝⊝ Very low quality: There is great uncertainty regarding the estimate. ^a^ Issues such as lack of blinding and unclear allocation criteria were identified. ^b^ The studies showed adequate methodological conduct, with no significant concerns. ^c^ The results were consistent across studies, with expected variations and no significant heterogeneity. ^d^ The evidence applies directly to the population, intervention, comparison, and outcome of interest. ^e^ The number of studies, events, or samples was limited, generating uncertainty in the confidence intervals and in the precision of the estimates. ^f^ The data were considered sufficient and robust, with acceptable confidence intervals.

## Data Availability

All data supporting the findings of this systematic review are available within the manuscript. In addition, coded data extraction forms and supplementary datasets generated during the review process are available from the corresponding author upon reasonable request.

## Supplementary Materials

The following supporting information can be downloaded at https://www.mdpi.com/article/10.3390/jcm15031310/s1, Table S1: PRISMA 2020 Checklist [32].
